# Heterologous prime-boost: breaking the protective immune response bottleneck of COVID-19 vaccine candidates

**DOI:** 10.1080/22221751.2021.1902245

**Published:** 2021-03-29

**Authors:** Qian He, Qunying Mao, Chaoqiang An, Jialu Zhang, Fan Gao, Lianlian Bian, Changgui Li, Zhenglun Liang, Miao Xu, Junzhi Wang

**Affiliations:** National Institutes for Food and Drug Control, Beijing, People’s Republic of China

**Keywords:** Heterologous prime-boost, COVID-19 vaccine, neutralizing antibody, T cell response, th1/th2 balance

## Abstract

COVID-19 vaccines emerging from different platforms differ in efficacy, duration of protection, and side effects. To maximize the benefits of vaccination, we explored the utility of employing a heterologous prime-boost strategy in which different combinations of the four types of leading COVID-19 vaccine candidates that are undergoing clinical trials in China were tested in a mouse model. Our results showed that sequential immunization with adenovirus vectored vaccine followed by inactivated/recombinant subunit/mRNA vaccine administration specifically increased levels of neutralizing antibodies and promoted the modulation of antibody responses to predominantly neutralizing antibodies. Moreover, a heterologous prime-boost regimen with an adenovirus vector vaccine also improved Th1-biased T cell responses. Our results provide new ideas for the development and application of COVID-19 vaccines to control the SARS-CoV-2 pandemic.

## Introduction

The mammoth scale of the outbreak of the COVID-19 pandemic has overwhelmed healthcare systems globally. Consequently, many countries worldwide have prioritized vaccine development for the containment of COVID-19. So far, there are 60 vaccine candidates in clinical trials, 13 of which are in the phase 3 stage. Although several leading COVID-19 vaccines have been shown to confer protection against SARS-CoV-2 infection [1–3], the emergence of novel SARS-CoV-2 variants [4,5], and the continuous decrease in the titres of antibodies in vaccinated individuals [6], raises public health concerns regarding the efficacy and duration of protection induced by the administration of such first-generation vaccines, which were developed rapidly for emergency use.

Currently, many COVID-19 vaccines developed for emergency use have their own advantages and disadvantages. For instance, mRNA vaccines such as BNT162b2 and mRNA-1273 have been shown to induce > 90% protection in the early stages of SARSCoV2 infection. However, the incidence of adverse reactions has raised concerns regarding the safety of such vaccines. Furthermore, stringent cold chain requirements for mRNA vaccines pose a significant logistical challenge [1,3,7]. Inactivated vaccines and recombinant protein-based vaccines, which are also part of the leading vaccine candidates, have exhibited lower incidences of adverse reactions. However, compared to mRNA vaccines, they exhibit inferior immunogenicity, even with the use of adjuvants [8–11]. T cell responses induced by the inactivated vaccine BBIBP-CorV [12] and recombinant vaccine ZF2001 [13] are relatively low. On the other hand, adenovectorbased vaccines, such as Ad5-vectored vaccine (CanSino), induce strong T cell responses, but only less-effective neutralizing antibody (NAb) responses than other approaches in humans [14]. These differences are likely to become more pronounced after the approval of such vaccine candidates for use in large populations.

The urgency for the development of a COVID-19 vaccine has led to a paradigm shift in process development, where many steps of vaccine development have been performed in parallel to save time. As a result, different types of COVID-19 vaccines emanating from multiple platforms and formulations [15] have progressed rapidly to advanced stages of development. In line with this paradigm shift, the availability of so many COVID-19 vaccine candidates could be tested for heterologous prime-boost vaccination strategies [16,17] to elicit higher and broader protective immune responses (both antibody and T cell responses), and for improved safety profiles. This exploration meets emergency needs and may assist in the formulation of public health policies.

## Materials and methods animals and vaccines

Specific pathogen-free BALB/c mice were provided and maintained by the Chinese National Institutes of Food and Drug Control. The four types of SARS-CoV-2 vaccines employed in this study included inactivated vaccines, adenovirus vectored vaccines, recombinant protein vaccines, and mRNA vaccines, and were donated by different manufacturers (Table 1).

**Table 1.** Information concerning vaccines used in this study.

Vaccines	Developer/Manufacturer	Platforms	Number of Doses	Human Dose	Ref. Clinical trials
BBIBP-CorV [8]	Beijing Institute of Biological Products/Sinopharm	Inactivated vaccine	2	4 μg	ChiCTR2000034780 NCT04560881
Ad5-nCoV [14]	CanSino Biological nc./Beijing Institute of Biotechnology	Adenovirus type 5 vectored vaccine	1	5 × 10^10^ vp	NCT04526990 NCT04540419
ZF2001 [13]	Anhui Zhifei Longcom Biopharmaceutical/Institute of Microbiology, Chinese Academy of Sciences	Recombinant RBD vaccine	2	50 μg	NCT04466085
ARcoVax	People's Liberation Army (PLA) Academy of Military Sciences/Walvax Biotech.	mRNA vaccine	2	25 μg	ChiCTR2000034112 ChiCTR2000039212

## ELISA for estimating total spike-specific IgG

Levels of serum binding antibodies directed against the SARS-CoV-2 spike protein were assessed using an ELISA-based assay. Ninety-six-well EIA/RIA plates were coated overnight with 1 µg/mL recombinant spike protein at 4°C. After removal of unbound spike protein and blocking with 10% fetal bovine serum in 0.5% PBST, 10-fold seriallydiluted test samples were added to the wells. Bound antibodies were subsequently detected after incubation with 1 : 5000 diluted goat anti-mouse IgG (HRP labeled) (China ZSGB-BIO, cat#ZB2305) followed by development with substrate (China Beijing Wantai BioPharm, cat#N20200722) at 450 nm and 630 nm. The endpoint of serum antibody titers was determined as the reciprocal of the highest dilution that was 2.1-fold higher than the optical absorbance value of the negative control.

## Serum neutralization assay

Levels of serum NAbs against SARS-CoV-2 were measured using live-and pseudoSARS-CoV-2 virus, and the results are expressed as geometric mean titers (GMT). NAbs against live SARS-CoV-2 (virus strain SARS-CoV-2/human/CHN/CN1/2020, GenBank: MT407649.1) were quantified using a microcytopathogenic effect assay at a minimum eight-fold dilution [11]. The neutralization capacity against pseudovirus (Wuhan-Hu-1, GenBank: MN908947, optimized for human cell expression) was determined following a previously reported protocol [18].

## IFN-γ ELISPOT assay

Freshly isolated splenocytes were stimulated with four separate peptide pools spanning the SARS-CoV-2 spike protein for 20 h at 2 × 10^5^ cells per well. The concentration of each peptide was 5 μg/mL. A total of four peptide pools were generated as follows: a panel of consecutive 15-mer peptides overlapping by 9 amino acids were synthesized to encompass the entire spike protein and grouped into four pools: S1-non RBD (aa:1–324, 577–654; 67 peptides), S1-RBD (aa:325–576; 42 peptides), S2-1 (aa: 655–960; 51 peptides), and S2-2 (aa:961-1273; 51 peptides). After stimulation, the plates were incubated with IFN-γ-detecting antibodies. Spots representing IFN-γ-producing cells were enumerated using an ELISPOT reader (ChampSpot III Elispot Reader, Saizhi, Beijing, China). Final determinations were calculated by subtracting background levels from measured values.

## MSD Th1/Th2 cytokine profiling

Freshly isolated splenocytes were stimulated with four peptide pools spanning the SARSCoV-2 spike protein for 20 h at 2 × 10^5^ cells per well. The concentration of each peptide was 5 μg/mL. Supernatants were collected and pooled according to different vaccination groups or different peptide pools. Supernatants were diluted 1:2 for unstimulated samples and then measured using a V-PLEX Proinflammatory Panel 1 (mouse) Kit. The concentration of each sample was calculated using a standard curve. The concentration of unstimulated samples was subtracted from the levels of stimulated samples.

## Statistical analysis

Antibody titers were transformed into log10 values to calculate geometric means and 95% confidence intervals (CIs). All statistical analyses were conducted using GraphPad Prism v.7.0 (GraphPad Software, Inc.). Comparisons between multiple groups were analyzed by one-way analysis of variance (ANOVA). Statistical significance was defined as *p*< 0.05.

## Results Humoral immune responses elicited by individual BBIBP-CorV, Ad5-nCoV, ZF2001, and ARcoVax vaccines

**Figure 1.**
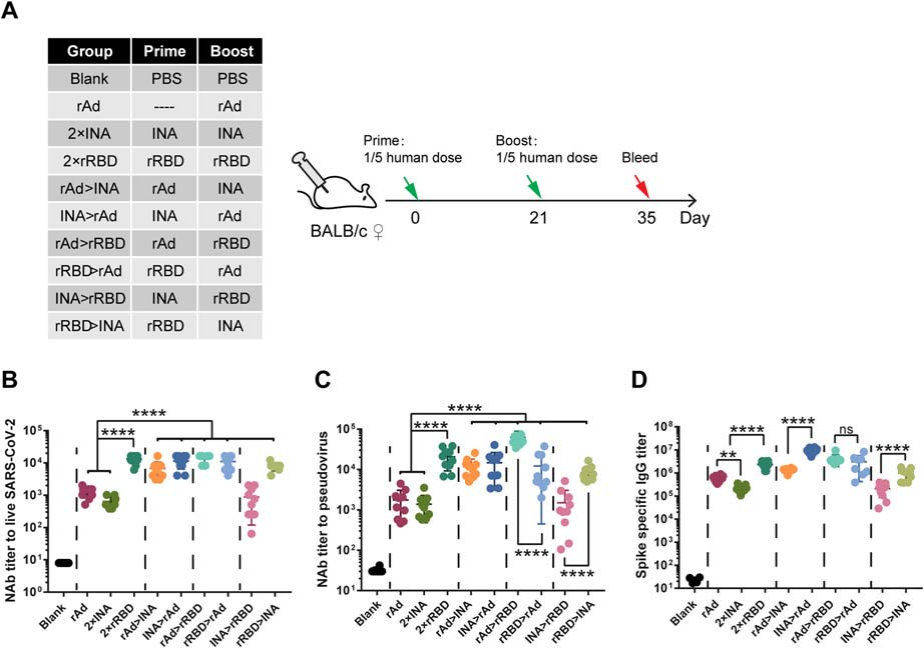
Comparison of humeral immune responses induced by COVID-19 vaccines of different technology platforms and heterologous prime-boost regimens. (A). Schematic representation of experimental protocols and immunization groups. Mice in 9 groups were immunized with different COVID-19 vaccines or vaccine combinations: rAd, 2 × INA, 2 × rRBD,, rAd > INA, INA > rAd, rAd > rRBD, rRBD > rAd, INA > rRBD, rRBD > INA. (rAd: recombinant Ad5 vectored vaccine, INA: inactivated vaccine, rRBD: recombinant RBD vaccine), Mice in a blank control group were sham-vaccinated with PBS. For rAd group, mice were immunized with one dose of rAd vaccine and blood samples were collected 14 days post -vaccination; for other groups, blood samples were only collected 14 days after the second vaccine dose (B,C). Serum Nab levels measured by live SARS-CoV-2 virus (B) and pseudovirus (C). NAb titres are expressed as 50% inhibitory dilution (EC50) of serum. D. Spike-specific binding IgG titres were measured by ELISA (n = 8–10 per group, one spot represents one sample). Bars represent means ± SD, **p < 0.01, ****p < 0.0001, ns: *p* > 0.05.

**Figure 2.**
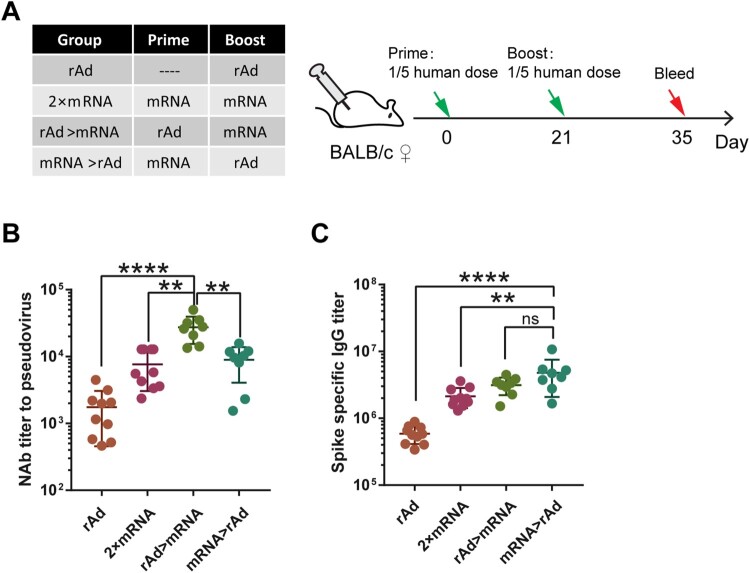
Humeral immune responses induced by heterologous prime-boost regimen of adenovirus vectored and mRNA-based COVID-19 vaccine. (A). Schematic representation of experimental protocol and immunization groups. Mice in 4 groups were immunized with adenovirus vectored vaccine or mRNA vaccine: rAd, 2 × mRNA, rAd > mRNA, mRNA > rAd. (rAd: recombinant Ad5 vectored vaccine, RNA: mRNAbased vaccine). For the rAd group, mice were immunized with one dose of rAd vaccine and blood samples were collected 14 days post-vaccination; for other groups, bloods were collected 14 days post the second vaccine dose. (B). NAbs of serum measured by live SARS-CoV-2 virus and expressed as 50% inhibitory dilution (EC50) of serum. (C). Spike-specific binding IgG titres were measured by ELISA (n = 8–10 per group, one spot represents one sample). Bars represent means ± SD, **p < 0.01, ****p < 0.0001, ns: *p* > 0.05.

Studies have shown that both humoral and cellular immune responses are related to the effectiveness of vaccines, of which the protective effect of NAbs is the most important [19]. For the first time in the current study, a unified evaluation of specific SARS-CoV2 binding and NAb titers elicited by individual Ad5-vectored vaccines (Ad5-nCoV: Cansino) (rAd), inactivated vaccine (BBIBP-CorV: Sinopharm) (INA), recombinant RBD vaccine (ZF2001: Zhifei) (rRBD), and mRNA vaccine (ARcoVax: Walvax) (mRNA) was performed (Table 1). The procedure followed the respective clinical immunization protocols of each vaccine, and uniformly 1/5th of the human dose was used to immunize mice (Table 1). The antibody levels of the mice were measured 14 days after the procedure (Figure 1A). For the rAd group, mice were immunized with one dose of rAd vaccine as in its human clinical use, and blood was collected 14 days post vaccination; for other groups, blood was collected only 14 days after the second dose. The results showed that the NAb GMT against live SARS-CoV-2 by rAd, INA, and rRBD vaccines were 1218, 596, and 12713, respectively (Figure 1B). Among these, the NAb titer induced by the rRBD vaccine was significantly higher than that induced by the other two vaccines (*p* < 0.0001), but there was no statistically significant difference between the NAb titers induced by the rAd vaccine and INA vaccine (*p* > 0.05). A similar trend was observed for the NAb GMT in an assay against pseudovirus and spike-specific binding antibody titers, as measured by ELISA (Figure 1B, C). The NAb GMTs against pseudovirus were 1348 in the rAd group, 1173 in the INA group, 17558 in the rRBD group (Figure 1C), and 6360 in the mRNA group (Figure 2B). Taken together, these results indicated that all types of vaccines tested in this study could induce good humoral immune responses in mice.

## Heterologous prime-boost immunization with adenovirus vectored vaccine, inactivated vaccine and recombinant subunit vaccine

To improve the protective immune responses of the above vaccines, we tested a twoby-two combination of rAd, INA, and rRBD vaccines using the heterologous prime-boost approach in the next mouse study. A dose of the vaccine consisting of 1/5th of the human dose used in clinical trials was used for each animal. The binding antibodies and NAb were compared with those elicited by the single vaccine type (Figure 1A).

Priming immunization with INA vaccine followed by rAd vaccine (INA>rAd) induced a 6.7-fold higher level of binding antibody titer than priming with rAd vaccine followed by INA (rAd>INA) (*p* < 0.0001) (Figure 1D). NAb levels in either rAd>INA or INA>rAd were significantly increased when compared with that of the single-type vaccine immunization design based on both live virus and pseudovirus assays (*p* < 0.0001). The NAb GMT induced by the rAd>INA group and the INA>rAd group were 5932 and 10206 against live SARS-CoV-2 and 10499 and 10932 80 against pseudovirus, respectively (Figure 1B, C).

For heterologous prime-boost immunizations using rAd and rRBD vaccines, the NAb GMT of the rAd>rRBD group and rRBD>rAd group were 13309 and 10033 based on live SARS-CoV-2, and 58604 and 8195 based on pseudovirus assays, respectively (Figure 1B, C). The NAb levels of rAd>rRBD and rRBD>rAd were significantly higher than those of the rAd vaccine alone (*p*  <  0.0001) (Figure 1B). For the live virus assay, there was no difference between the 2 × rRBD, rAd>rRBD, and rRBD>rAd groups. However, for the pseudovirus assay, NAb GMT of rAd prime-rRBD boost (rAd>rRBD) was significantly higher than that of the 2 × rRBD vaccine (*p*  =  0.0002). Interestingly, although both rAd>rRBD and rRBD>rAd groups induced comparable levels of binding antibodies, the GMT of NAbs induced by the rAd>rRBD group against pseudovirus was 7.15-fold higher than that of the rRBD>rAd group (*p*  <  0.0001) (Figure 1C, D).

We then compared the heterologous prime-boost approach with INA and rRBD vaccines. The NAb GMT of “rRBD>INA” group was 7625 based on live SARS-CoV-2 virus assay, which was 12.8-fold higher than that of the INA vaccine alone (*p*  <  0.0001), and significantly higher than that of the “INA>rRBD” group (*p*  <  0.0001) (Figure 1B). A similar difference was found for the pseudovirus-based assay and binding antibodies (Figure 1C, D).

## Heterologous prime-boost immunization with adenovirus vaccine and mRNA vaccine

Next, we performed prime-boost immunizations with the rAd vaccine and mRNA vaccine (Figure 2A). 1/5th of the human dose was used in this experiment (Table 1). Antibody levels were measured 14 days after immunization. The GMT of the rAd > mRNA and mRNA > rAd groups was compared with the rAd group and the 2 × mRNA group. Similar to what we observed above, the rAd vaccine prime, followed by an mRNA vaccine boost (rAd > mRNA), induced a significantly higher NAb response than the 2 × mRNA vaccine, with a GMT of 25,186 (*p* < 0.01) in the pseudovirus assay (Figure 2B). Although the amount of binding antibody induced by mRNA > rAd was comparable to that induced by the rAd > mRNA (Figure 2C), the NAb response was significantly lower than rAd > mRNA (*p* < 0.01).

## T cell responses elicited by heterologous prime-boost immunization using adenovirus vaccine followed by inactivated/recombinant subunit vaccine

**Figure 3.**
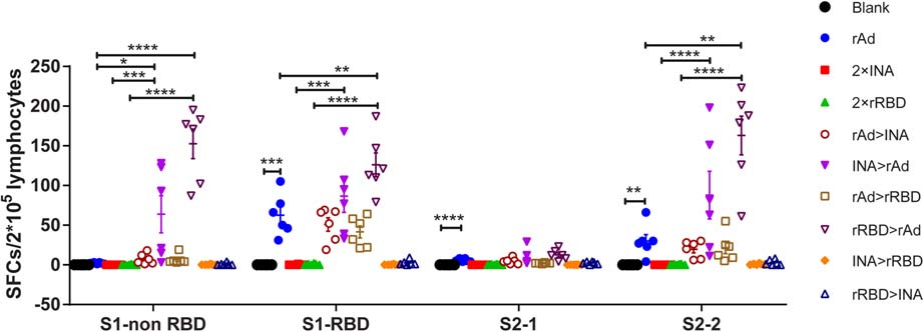
T cell responses to SARS-CoV-2 spike peptides measured by IFN-γ ELISPOT. 6 mice in Figure 1 were sacrificed and T cell response were measured. Isolated lymphocytes were stimulated with 4 peptide pools spanning spike respectively, and the IFN-γ secreting cells were quantified by ELISPOT assay. (n = 6 per group, one spot represents one sample). Bars represent means ± SEM, *p < 0.05, **p < 0.01, ***p < 0.001, ****p < 0.0001.

To investigate S-antigen-specific T cell responses induced by different regimens (Figure 1), splenic lymphocytes were collected and stimulated with four peptide pools spanning the SARS-CoV-2 spike protein for 20 h and IFN-γ ELISPOT assays were conducted. Our results showed that the most recognized peptide pools were S1-RBD (aa: 325–576) and S2-2 (aa: 961–1273). For the single vaccine regimen, only the rAd group induced an obvious magnitude of T cell responses of 62.5% SFUs (Spot Forming Units) against S1RBD and 30 SFUs against S2-2 per 2 × 10^5^ splenic lymphocytes, while the T cell responses in the 2 × INA and 2 × rRBD groups were quite low or even undetectable. Interestingly, when the rAd vaccine was combined with the INA or rRBD vaccine, T cell responses were further amplified. INA > rAd (S1-RBD: 86.5, S2-2: 87.3) induced a higher level of T cell response than rAd > INA (S1-RBD: 50.8, S2-2:19.5), and levels in both groups were significantly higher than 2 × INA (all *p* < 0.05). Similar to 2 × INA, T cell responses induced by 2 × rRBD were also amplified against all four peptide pools when primed or boosted with an adenovirus vector vaccine. rRBD > rAd induced higher levels of T cell responses than all other groups, with SFUs of 152.5, 126.1, 12.7, and 163 against S1-non RBD, S1-RBD, 12.7 against S2-1, and 163 against S2-2. Although the rAd > rRBD regimen induced lower T cell responses than rRBD > rAd, the magnitude of T cell responses induced by rAd > rRBD was still significantly higher than that in the 2 × rRBD group (S1-RBD, *p* = 0.003) (Figure 3).

## Th subtype analysis after heterologous prime-boost immunization using adenovirus vaccine followed by inactivated/recombinant subunit vaccine

**Figure 4.**
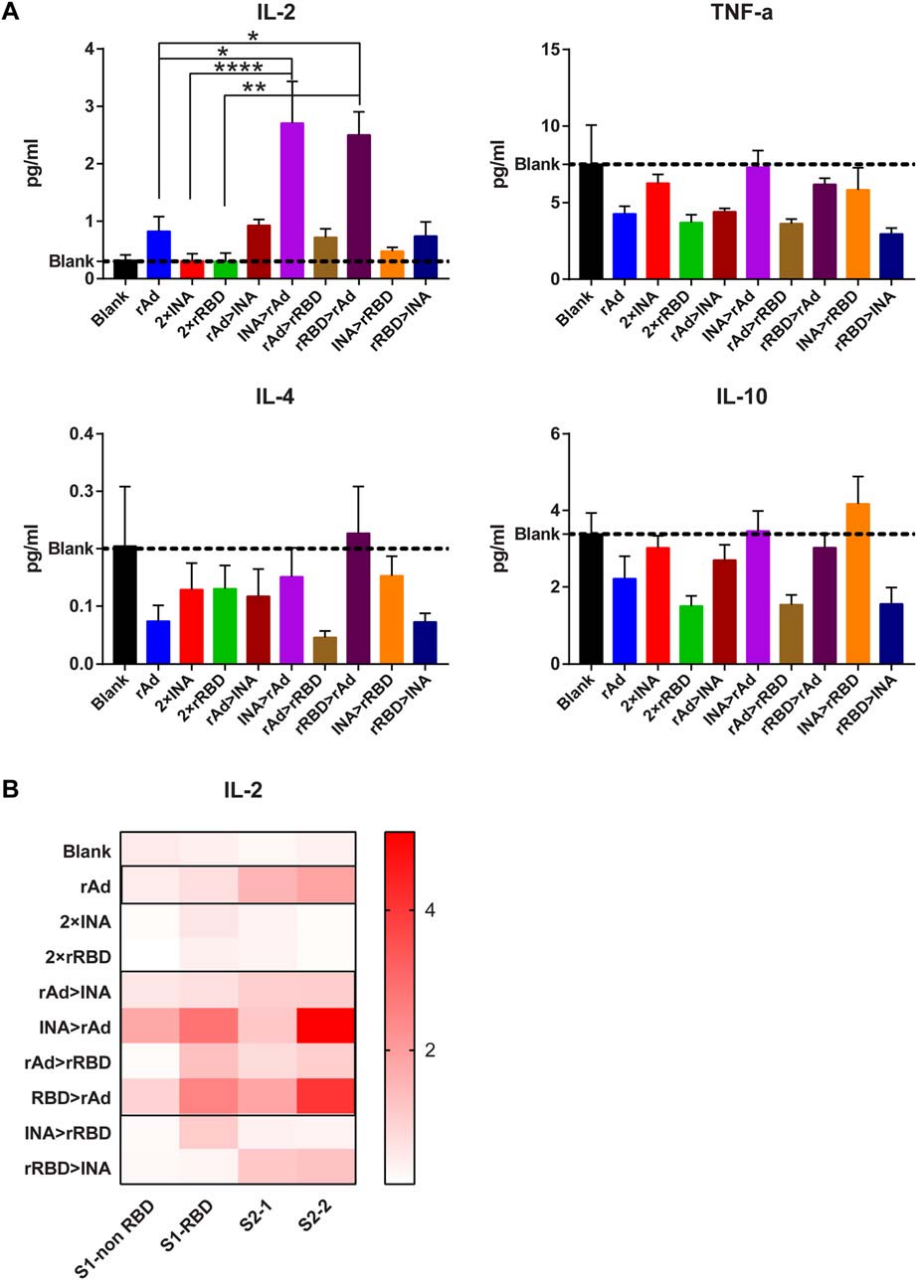
Multiplex cytokine analysis for different immunization regimens. Isolated lymphocytes in 9 regimens identified in Figure 1 were stimulated with 4 spike peptide pools. Supernatants pooled by different groups (A) or different peptide pools (B) were collected. IL-2, IL-4, IL-10 and TNF-α levels in supernatants were measured by MSD; the concentration of each cytokine(pg/mL) is represented by histogram (A) or heatmap (B) (n = 6 per group). Bars represent means ± SEM, *p < 0.05, **p < 0.01, ****p < 0.0001.

To further investigate whether the heterologous prime-boost regimen induces a Th1 biased T cell immune response, we collected the supernatant of splenic lymphocytes stimulated by spike peptides (Figure 3) and pooled the supernatants according to different groups or different peptide pools. Th1/Th2 cytokines in supernatants were measured using the Meso Scale Discovery Assay (MSD). Since IFN-γ had already been bound to the plate for ELISPOT assays, we only measured IL-2/TNF-α for Th1 cytokines and IL4/IL-10 for Th2 cytokines. IL-4 and IL-10 levels were lower or comparable in all vaccination groups compared to the blank control; thus, Th2 cytokine levels did not increase post-vaccination (Figure 4A). Antigen-specific IL-2 cytokine was only detectable in the rAd group among the three single vaccine regimens compared with the blank control, with a concentration of 0.83 pg/mL. Moreover, IL-2 levels were elevated in INA > rAd and rRBD > rAd groups, with mean concentration of 2.7 and 2.5 pg/mL, respectively. A modest increase in IL-2 abundance was measured in the rAd > INA and rAd > rRBD groups compared with the 2 × INA or 2 × rRBD regimens (Figure 4A). The intensity of the IL-2 response stimulated by the four different peptide pools was analyzed and represented as a heatmap, which showed that S1-RBD and S2-2 peptide pools were the most recognized and IL-2 levels against all four peptide pools were relatively elevated in the 2 × rAd, rAd > INA, INA > rAd, rAd > rRBD, and rRBD > rAd groups (Figure 4B). Multiple cytokine analysis indicated that the rAd vaccine could induce superior T cell responses, and heterologous prime-boost with adenovirus vectored vaccine and inactivated/recombinant subunit vaccine might mediate a bias toward secreting Th1 cytokines by splenocytes stimulated with SARS-CoV-2 spike peptides.

## Discussion

Heterologous prime-boost strategies have been widely used for the administration of HIV vaccines. Previous studies have shown that prime-boost immunization with heterologous vaccines can increase the intensity and breadth of immune responses.

However, to date, there have been no studies concerning the heterologous prime-boost strategy for COVID-19 vaccines. In this study, compared with single-dose COVID-19 vaccine immunization, priming with Ad5-vectored vaccines followed by inactivated vaccines, recombinant protein, or mRNA vaccines significantly improved the NAb response profile, which is consistent with the conclusions of previous research on heterologous prime-boost strategies [16,20,21]. The enhanced NAb titres could be attributed to the heterologous prime-boost strategy, which probably leads to reduced antivector and anti-non-spike protein immune responses, generating higher levels of antispike protein immune responses. Furthermore, heterologous prime-boost strategies with mRNA and Ad5-vectored vaccines were evaluated in parallel for the first time in this study. Our results indicated that “Ad5-vectored vaccine prime-mRNA vaccine boost” can improve NAb levels and can be more effective than a “mRNA vaccine prime-Ad5vectored vaccine boost” regimen. We still do not know why the two types of vaccines that express antigens in the cytoplasm elicit different immune responses when sequentially inoculated. We speculate that this may be related to the different natural immune responses activated by the delivery system. A thorough study of the mechanisms underlying immune responses induced by adenovirus vectored and mRNA vaccines is likely to shed more light on this intriguing difference surrounding the capabilities of the two different types of vaccines.

NAb titres are highly correlated with the protective effects and durability of such protection. The results of previous studies involving monoclonal antibodies and convalescent sera, as well as tests conducted in animal models, have confirmed the role of NAbs in conferring protection against COVID-19 [19,22,23]. Recently, an initial analysis by our group showed that the efficacy of current vaccine candidates is related to the levels of GMT of NAbs to some degree (unpublished data). However, the immunogenicity and safety of the currently developed vaccines are imbalanced. BBIBPCorV, an inactivated vaccine investigated in this study, possesses a good safety profile but relatively low immunogenicity; mRNA vaccines, such as BNT162b2 and mRNA1273, showed greater immunogenicity but relatively higher adverse reaction rates [1,3,8,24,25]. A sequential immunization strategy using heterogeneous vaccines could potentially improve the protective efficacy of inactivated vaccines and provide a viable solution for mitigating the adverse effects of mRNA vaccines, especially those caused by second doses.

When Ad5-vectored vaccines were used as primary vaccines, relatively high levels of NAbs could be induced while maintaining the level of binding antibodies, indicating that the proportion of NAbs in the humoral immune response increased. Studies involving dengue virus (DENV) and MERS suggested that binding antibodies could mediate viral entry into cells, leading to antibody-dependent enhancement (ADE) [26,27]. We should be alert to the potential safety issues surrounding the immune response state of “low NAbs and high binding antibodies” Our data suggested that a heterologous prime-boost strategy with existing COVID-19 vaccines of different technology platforms may help solve these issues arising from the lower levels of protective NAbs when compared to binding antibodies elicited by certain vaccines.

Effective activation of T cell responses after vaccination is an outstanding feature of adenovirus-vectored vaccines, which may be attributed to cytoplasmic antigen expression (Figures 3 and 4)[7,14,28]. Although our data revealed that the adenovirus vectored vaccine induced a significantly higher T cell response activation potential (as indicated by IFN-γ and IL-2 abundance) than inactivated or recombinant RBD vaccine. In addition, the Th1 biased T cell response elicited by a single dose of adenovirus vectored vaccine can be further amplified when primed by inactivated or recombinant RBD vaccine, but not boosted by the two other types of vaccines. Interestingly, the proportion of NAbs exhibited a different trend in that the titre was higher when adenovirus vectored vaccine was used as a primary vaccine and inactivated or recombined RBD vaccine as a booster innoculation. We speculate that the activation and differentiation of T cell responses (such as follicular helper T cells) [29–31] induced at an early stage by a primary vaccine may improve the effect of the booster vaccine, probably by regulating antibody formation and balancing the production of protective NAbs and binding antibodies [32]. For this consideration, the dynamics of follicular helper T cell responses should be further investigated to explain such a phenomenon, as found in this study.

In summary, this study investigated four COVID-19 candidate vaccines undergoing clinical trials for a prime-boost regimen in a mouse model. The heterologous prime-boost strategy improved the levels of NAbs and Th1 biased T cell responses. Interestingly, “adenovirus vectored vaccine as a primary vaccine, inactivated/recombinant subunit/mRNA vaccine as booster” promoted the modulation of antibody responses to NAbs. It should be noted that these experiments were limited to measuring immune responses and cannot be directly translated to levels of protection. The durability of immune responses needs to be further assessed, which may vary depending on the specific combination of vaccines employed. Finally, murine data do not always translate to humans, and similar trials in humans are needed to confirm the results described here. Our results provide new ideas for the development and application of COVID-19 vaccines to control the SARS-CoV-2 pandemic.

## Data and materials availability

All data generated in this study are available from the corresponding author upon reasonable request. Pseudoviruses used for NAb measurements can be obtained from the Chinese National Institution for Food and Drug Control. The live SARS-CoV-2 used fo NAb measurements was isolated by Sinovac Research and Development Co., Ltd.
